# 폐경 후 부인암 환자의 수술 전 노쇠와 수술 후 건강 결과: 후향적 단면 조사 연구

**DOI:** 10.7586/jkbns.25.053

**Published:** 2025-11-11

**Authors:** Huijin Kim, Wonhee Baek

**Affiliations:** 1 Gyeongsang National University Hospital, Jinju, Korea; 1 경상국립대학교병원; 2 College of Nursing, Gyeongsang National University, Jinju, Korea; 2 경상국립대학교 간호대학 간호학과

**Keywords:** Frailty, Genital neoplasms, Female, Postoperative complications, Postmenopause, 노쇠, 부인암, 수술후 합병증, 폐경후

## Abstract

**Purpose:**

This study aimed to assess preoperative frailty using the Frailty Index–Laboratory (FI-LAB) in postmenopausal patients undergoing gynecological cancer surgery and to investigate the association between frailty level and postoperative health outcomes.

**Methods:**

A retrospective review was conducted of medical records from 198 postmenopausal women who underwent gynecologic cancer surgery at Gyeongsang National University Hospital between January 2018 and December 2024. Frailty was evaluated using the FI-LAB, and postoperative outcomes included recovery room stay, hospital stay, transfusion, and complications. Data were analyzed using analysis of variance and the chi-square test.

**Results:**

Higher frailty levels were associated with older age (*p* = .030), cancer type (*p* < .001) and stage (*p* = .001), American Society of Anesthesiologists Physical Status Classification class (*p* = .036), and longer anesthesia (*p* = .023) and surgery time (*p* = .026). Among health outcomes, patients with higher frailty scores had longer hospital stays (F = 4.26, *p* = .015) and higher transfusion rates (χ^2^ = 11.78, *p* = .005).

**Conclusion:**

Preoperative frailty assessed by FI-LAB may serve as a useful predictor of postoperative outcomes in patients undergoing gynecologic cancer surgery. Early identification of frailty could enable targeted perioperative interventions to improve recovery and support the integration of frailty screening into routine preoperative evaluation.

## 서론

### 1. 연구의 필요성

폐경은 여성의 생식 기능이 종결되는 자연스러운 생리적 변화로, 국내 여성의 평균 폐경 연령은 49.2세로 보고된다. 여성의 기대수명이 85.6세인 점을 감안할 때, 여성은 생애의 약 1/3 이상을 폐경 이후 시기로 보내게 된다[1,2]. 폐경 이후의 에스트로겐 분비 감소는 근골격계 기능 약화, 대사 항상성 저하 등을 생리적 변화를 초래하여 전반적인 건강 상태에 영향을 미친다[3,4].

부인암은 전 세계적으로 발병률이 지속적으로 증가하고 있으며[5], 2022년 통계에 따르면 국내에서 자궁체부암은 3,958명, 난소암은 3,263명 발생하여 여성암 발생률 중 8위와 10위를 차지하였다. 1993~1995년 대비 2018~2022년 동안 여성 암종의 상대생존율 추이를 살펴보면, 폐암(39.0%)과 위암(33.3%)의 상대생존율은 현저히 증가하였으나, 자궁체부암(6.1%)과 난소암(5.6%)은 여전히 낮은 생존율을 보이고 있다[6,7]. 따라서 폐경기 부인암 환자의 회복과 생존율을 향상시키기 위해서는 건강결과 향상에 기여하는 관련 요인을 이해하고, 이를 바탕으로 효과적인 치료 전략을 마련하는 것이 필요하다.

에스트로겐은 항산화 작용과 면역조절 기능을 통해 세포 손상과 염증 반응을 억제한다. 그러나 폐경 이후 이러한 효과가 줄어들면서 생리적 항상성이 저하되고, 이는 생리적 예비력이 감소한 상태를 의미하는 노쇠 발생의 중요한 기전으로 작용한다[8-10]. 에스트로겐 감소에 따른 대사적 불균형과 만성 염증은 부인암 발생 위험과도 연관성이 있다는 점에서[11], 폐경 후 여성은 암 발생과 노쇠가 동시에 증가할 수 있는 생물학적 취약성을 가지게 된다.

노쇠한 환자는 스트레스 상황에 대한 대응 능력이 저하되어 건강 악화 및 부정적 예후의 위험이 증가한다[12]. 암 환자의 노쇠 유병률은 환자 집단의 특성 및 노쇠 평가 도구의 차이에 따라 5%~90%까지 다양한 유병률 범위를 보인다[13]. 이와 비교할 때, 부인암 환자의 노쇠 유병률은 6.9%에서 33.3%까지 보고되어 전체 암 환자에서 보고된 범위의 하위~중간 수준에 해당한다. 폐경 후 암환자의 생리학적 특성을 고려할 때 상당수 환자가 노쇠 상태일 가능성이 높다[14-16]. 암 환자에서 노쇠는 수술, 항암화학요법, 방사선치료 등 치료 전반에 영향을 미치는 중요한 예후 인자로 작용하며, 수술 후 합병증, 장애, 사망 등의 발생 위험과도 밀접한 관련이 있다[17-19]. 따라서, 부인암 환자의 회복과 건강 결과 향상에 주요 관련 요인으로 수술 전 노쇠 상태가 고려될 수 있다.

노쇠를 평가하는 방법으로는 노쇠 표현형(Frailty Phenotype)과 노쇠 지수(Frailty Index)가 있다. 노쇠 표현형은 신체 수행 능력에 중점을 둔 평가 방식으로, 의도하지 않은 체중 감소, 악력 감소, 자가보고한 탈진, 보행 속도 저하, 낮은 신체 활동 수준의 다섯 가지 기준 중 세 가지 이상을 충족한 경우 노쇠로 정의된다[12]. 한편, 노쇠 지수는 노쇠와 관련된 건강 문제와 개인의 전반적인 건강 상태 간의 비율로 정의된다[20]. 노쇠 지수 중 하나인 FI-LAB (Frailty Index-Laboratory)은 검체 검사 결과와 활력징후를 기반으로 산출된다[21]. FI-LAB은 전자의무기록(Electronic Medical Record)을 통해 산출이 가능하여 평가에 소요되는 시간을 단축할 수 있고 모든 환자에게 자동적으로 적용할 수 있으므로 임상 현장에서 실용적으로 활용될 수 있다[21].

따라서 본 연구는 폐경 후 부인암 수술 환자의 수술 전 노쇠 수준을 FI-LAB을 통해 평가하고, 노쇠 수준에 따른 수술 후 건강 결과의 차이를 분석함으로써, 노쇠 지수를 활용한 고위험군 조기 선별 및 임상 간호중재에 활용 가능한 근거를 마련하고자 한다.

### 2. 연구의 목적

본 연구의 목적은 폐경 후 부인암 수술 환자의 수술 전 노쇠와 수술 후 건강 결과의 관련성을 확인하는 것이며, 구체적인 목적은 다음과 같다.

1) 대상자의 일반적 특성 및 임상적 특성을 파악한다.

2) 대상자의 수술 전 노쇠 수준을 파악하고, 수술 전 노쇠 수준에 따른 일반적 특성 및 임상적 특성의 차이를 확인한다.

3) 대상자의 수술 전 노쇠 수준에 따른 수술 후 건강 결과의 차이를 파악한다.

## 연구 방법

### 1. 연구 설계

본 연구는 전자의무기록을 이용하여 폐경 후 부인암 수술 환자의 수술 전 노쇠와 수술 후 건강 결과를 확인하기 위한 후향적 조사 연구이다.

### 2. 연구 대상

본 연구는 경상남도 진주시에 소재한 상급종합병원인 경상국립대학교병원 산부인과에 입원하여 부인암 수술을 받은 폐경 후 여성 환자를 대상으로 하였다. 자료 수집 기간은 2018년 1월 1일부터 2024년 12월 31일까지였으며, 연구의 일관성을 유지하고 대상자의 임상적 동질성을 확보하기 위하여 다음과 같은 기준에 따라 연구대상을 선정하고 제외하였다.

**Figure 1.**
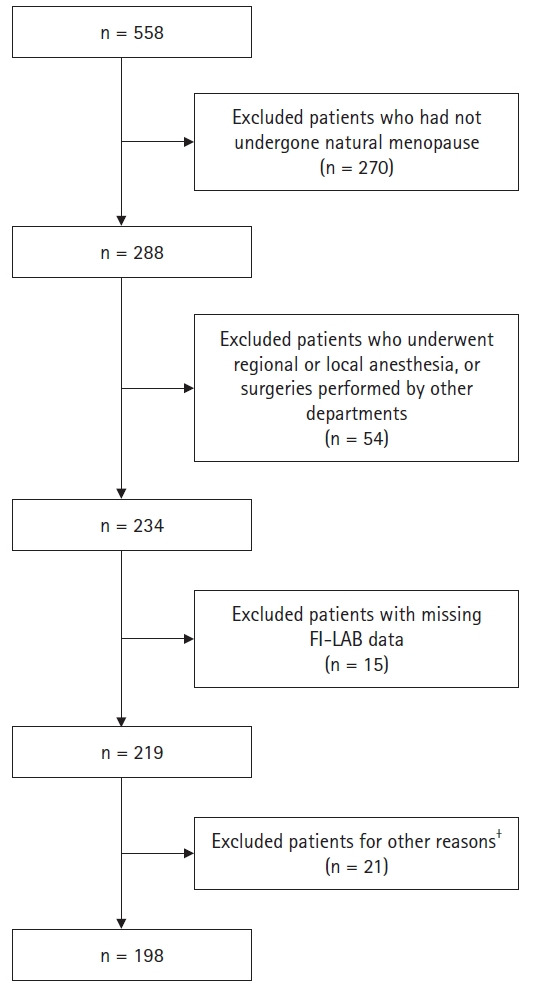
Flow diagram of study population. FI-LAB = Frailty Index-Laboratory. ^†^This category includes patients who underwent emergency surgeries (n = 9), cases excluded due to scheduling errors (n = 9), and patients admitted to the intensive care unit (n = 3).

선정기준은 자궁경부암, 자궁체부암, 난소암, 질암, 외음부암으로 진단받고 해당 부인암에 대한 수술을 받은 폐경 후 여성이었다. 제외기준은 자연 폐경이 아닌 환자(n = 270), 부인과 수술이 아닌 수술을 받은 환자 및 국소 또는 부위 마취로 수술을 받은 환자(n = 54), FI-LAB 항목 32개 중 결측 항목이 9개 이상인 환자(n = 15), 응급 수술을 받은 환자, 전자의무기록상 수술명이 중복 기재된 환자, 수술 직후 중환자실에 입원한 환자(n = 21)였다. 최종적으로 선정기준에 해당하는 558명 중 제외 기준에 해당하는 360 명을 제외한 198명이 본 연구의 최종 분석 대상자가 되었다(Figure 1). G*Power를 이용하여 효과크기 f = 0.25, 유의수준 α = .05, 검정력(1-β) = .80으로 산출한 결과 최소 필요 표본 수는 158명으로, 본 연구의 최종 표본 수 198명은 이를 충족하였다.

### 3. 연구 도구

본 연구는 전자의무기록을 활용한 이차자료분석 연구로 사용된 데이터의 출처는 Appendix A에 제시되었다.

#### 1) 노쇠

본 연구에서 노쇠는 FI-LAB을 산출하여 평가하였다[21]. FI-LAB은 수술 직전 시행된 27개의 검사 결과와 입원 직후 측정한 5개의 활력징후를 포함한 총 32개 항목으로 구성된다. 각 항목은Fried 등[22]의 분류에 따라 빈혈(Anemia), 염증(Inflammation), 내분비(Endocrine), 영양소(Nutrients), 비만(Adiposity), 운동(Motor), 기타(Others)의 범주로 분류하였다(Appendix B).

FI-LAB 산출 시 32개 항목 중 결측치가 9개 이상(30% 이상) 존재할 경우 신뢰성 확보를 위해 분석에서 제외하였다[21]. 본 연구에서 결측치는 주로 Folate (serum), Vitamin B12, Vitamin D 항목에서 발생하였다. 각 항목은 검사 수치가 정상 범위를 벗어나는 경우 1점(비정상 결과), 정상 범위일 경우 0점으로 부여되며, FI-LAB 점수는 결측치를 제외한 전체 항목 수 중 비정상 결과의 비율로 계산된다. 예를 들어, 31개 항목 중 10개가 비정상 결과일 경우 FI-LAB 점수는 0.32로 산출된다. 점수 범위는 0~1점이며, 점수가 높을수록 노쇠 수준이 높은 것으로 해석한다[21,23]. 본 연구에서는 전체 암 수술 환자를 대상으로 FI-LAB 과 수술 후 건강결과의 관련성을 확인한 Kim 등[24]의 연구를 근거로 노쇠 수준을 분류하였다. FI-LAB 점수가 0.25 미만은 낮은 노쇠, 0.25 이상 0.4 이하를 중등도 노쇠, 0.4를 초과하는 경우를 높은 노쇠로 정의하였다.

#### 2) 수술 후 건강 결과

건강 결과는 증상 상태, 기능 상태, 정서 상태, 사망률, 이환율 및 합병 질환, 삶의 질, 비용, 자가 관리를 의미한다[25]. 본 연구에서 수술 후 건강 결과는 회복실 체류시간, 수술 후 재원 기간, 수혈 유무, 수술 후 합병증의 4가지 지표를 사용하여 정의하였다.

회복실 체류시간은 회복실 간호기록지를 기준으로 회복실 입실 시점부터 퇴실 시점까지의 시간을 분 단위로 산출하였다. 수술 후 재원 기간은 수술일로부터 퇴원일까지의 일수로 계산하였다. 수혈 유무는 수술일부터 퇴원일까지의 기간 중 수혈이 시행된 경우로 정의하였다.

수술 후 합병증은 수술 부위 감염, 요로 감염, 요정체, 폐렴, 섬망의 다섯 가지 항목을 포함하였으며, 각 항목은 ‘있음’과 ‘없음’으로 구분하여 이분형 변수로 처리하였다. 수술 부위 감염은 수술일부터 퇴원일까지 해당 감염에 대한 의무기록이 있는 경우로 정의하였다. 요로 감염은 임상 증상이 나타나거나 소변 배양 검사에서 병원체가 검출되었으며, 이에 대한 치료나 간호중재가 시행된 경우로 간주하였다. 요정체는 자연배뇨가 되지 않거나, 배뇨 후 잔뇨량이 많은 상태로, 간헐적 도뇨가 시행되었거나 유치도뇨관이 재삽입된 경우, 혹은 자연배뇨 후 잔뇨량이 200cc 이상인 경우가 2회 이상 발생한 경우로 정의하였다. 폐렴은 해당 기간 내 폐렴 진단이 기록되어 있고, 이에 따른 의학적 중재가 시행된 경우로 간주하였다. 섬망은 시간, 장소, 사람에 대한 지남력 저하 또는 의식 수준의 변화가 의무기록에 명시되었거나, 정신건강의학과에 의뢰된 경우로 정의하였다. 위의 다섯 가지 중 하나 이상의 합병증이 발생했을 경우 합병증 있음으로 분류하였다.

### 4. 자료 수집

본 연구의 자료는 의무 기록 관련 부서 협조를 통해 2018년 1월 1일부터 2024년 12월 31일까지 부인암 수술을 받은 폐경 후 여성 환자의 입원 기록과 간호 정보 조사지, 임상관찰 기록지, 영양검색 상태지, 수술기록지, 마취기록지, 회복실 간호기록지, 검체 검사 결과지, 수혈처방 기록지, 영상검사 결과지, 퇴원 기록지를 포함한 의무기록을 확인하였다.

### 5. 자료 분석

자료 분석은 R 프로그램(version 4.0.3, R Foundation for Statistical Computing, Vienna, Austria)을 이용하여 다음과 같은 통계적 처리과정을 거쳐 분석을 시행하였다.

1) 대상자의 일반적 특성과 임상적 특성, FI-LAB으로 계산한 노쇠 점수, 수술 후 건강 결과는 변수의 특성에 따라 평균과 표준편차, 빈도와 백분율을 이용하여 기술하였다.

2) 대상자의 수술 전 노쇠에 따른 일반적 특성 및 임상적 특성의 차이는 변수의 특성에 따라 chi-square test, analysis of variance (ANOVA)를 이용하여 분석하고 Tukey’s honestly significant difference (HSD) test로 사후 검정을 시행하였다.

3) 대상자의 수술 전 노쇠 수준에 따른 수술 후 건강 결과는 변수의 특성에 따라 chi-square test, ANOVA를 이용하여 분석하고 Tukey’s HSD test, Bonferroni로 사후 검정을 시행하였다.

### 6. 윤리적 고려

본 연구는 경상국립대학교병원 생명윤리 위원회의 연구 승인(승인번호: GNUH 2025-02-008-001)을 받아 연구를 진행하였다. 수집된 자료는 개인 신상정보를 식별할 수 없도록 연구 코드를 부여하여 관리하였다. 연구를 통해 확보된 의무기록은 파일형태로 암호화하여 연구자의 개인 노트북에 보관하였으며, 연구 자료들은 연구 종료 후 3년간 보관한 후 파기할 것이다.

## 연구 결과

### 1. 대상자의 일반적 특성 및 임상적 특성

전체 198명의 대상자의 평균 연령은 62.07 ± 8.49세였으며, 65세 미만이 133명(67.2%)으로 가장 많았다. 폐경 연령의 평균은 51.38 ± 4.42세였고, 폐경 후 경과 기간은 평균 10.15 ± 8.25년이었다. 체질량지수는 평균 24.33 ± 4.02 kg/m^2^였으며, 비만에 해당하는 대상자는 79명(39.9%)이었다. 영양 상태는 저위험군이 115명(58.1%)으로 가장 많았다. 부인암 종류는 자궁체부암이 85명(42.9%), 난소암 62명(31.3%), 자궁경부암 49명(24.8%), 질암 2명(1.0%) 순이었다. 병기는 Ⅰ기 환자가 139명(70.2%)으로 가장 많았다. 수술 전 항암화학요법을 받은 대상자는 12명(6.1%)이었고, ASA class (American Society of Anesthesiologists Physical Status Classification)은 Ⅱ등급이 148명(74.7%)으로 가장 많았다. 마취 시간은 평균 204.06 ± 83.56분, 수술 시간은 평균 167.29 ± 80.38분이었다. 수술 방법은 복강경 수술이 86명(43.4%)으로 가장 많았고, 개복 수술이 75명(37.9%), 로봇 수술이 37명(18.7%)이었다(Table 1).

### 2. 대상자의 노쇠 수준

FI-LAB을 통해 산출한 수술 전 노쇠 지수는 평균 0.25 ± 0.10이었으며, 낮은 노쇠 수준(0.25 미만)에 해당하는 대상자는 103명(52.0%), 중등도 노쇠 수준(0.25–0.4)은 79명(39.9%), 높은 노쇠 수준(0.4 초과)은 16명(8.1%)이었다(Table 2).

### 3. 대상자의 노쇠 수준에 따른 일반적 및 임상적 특성 비교

일반적 특성에서 연령은 노쇠 수준에 따라 유의한 차이를 보였으며, 높은 노쇠 집단의 평균 연령이 낮은 노쇠 집단에 비해 유의하게 높았으나(F = 3.56, *p* = .030), 65세 이상과 미만으로 구분하였을 때는 노쇠 수준 간 유의한 차이가 나타나지 않았다.

임상적 특성에서는 부인암 종류에 따라 노쇠 수준에 유의한 차이가 나타났다(χ^2^ = 19.41, *p* < .001). ASA class에서도 유의한 차이가 나타났으며(χ^2^ = 12.12, *p* = .036), 마취 시간은 노쇠 수준에 따라 유의한 차이가 있었고, 높은 노쇠 집단이 낮은 노쇠 집단보다 유의하게 길었다(F = 3.85, *p* = .023). 수술 시간 또한 노쇠 수준에 따라 유의한 차이가 있었다(F = 3.70, *p* = .026) (Table 3).

### 4. 노쇠 수준에 따른 수술 후 건강 결과 비교

전체 대상자의 평균 회복실 체류시간은 33.17 ± 8.44분이었으며, 수술 후 재원 기간은 평균 13.47 ± 8.96일이었다. 수술 후 수혈을 받은 대상자는 30명(15.2%)이었고, 수술 후 합병증이 발생한 대상자는 28명(14.1%)이었다.

노쇠 수준에 따른 비교 결과, 수술 후 재원 기간은 노쇠 수준에 따라 유의한 차이를 보였으며(F = 4.26, *p* = .015), 높은 노쇠 집단이 낮은 노쇠 집단보다 재원 기간이 유의하게 길었다. 또한, 수술 후 수혈 유무는 노쇠 수준에 따라 유의한 차이를 보였고(χ^2^ = 11.78, *p* = .005), 높은 노쇠 집단에서 수혈을 받은 대상자의 비율이 낮은 노쇠 집단에 비해 유의하게 높았다(Table 4).

## 논의

본 연구는 부인암 수술을 받은 폐경 후 여성 198명을 대상으로 수술 전 노쇠 수준을 의무기록 기반 FI-LAB으로 평가하고, 노쇠 수준에 따라 대상자의 일반적 및 임상적 특성과 수술 후 건강 결과의 차이를 분석하였다. 부인암 환자의 특성과 연관성을 근거로, 수술 전 FI-LAB기반 노쇠 측정의 타당성을 검증하고, 이를 통해 향후 맞춤형 간호중재 및 수술 예후 예측을 위한 기초 자료를 제시하고자 하였다.

본 연구에서 FI-LAB을 기반으로 산출한 대상자의 평균 노쇠 지수는 0.25 ± 0.10이었으며, 높은 노쇠 수준(FI > 0.4) 대상자가 8.1%로 확인되었다. 이는 Kim 등[24]의 다기관 대규모 암 수술 환자를 대상으로 한 연구에서 보고된 높은 노쇠 수준 비율(4.0%)보다 높은 수치로, 이러한 차이는 연구 대상자의 암 종류, 병기, 진료 환경, FI-LAB 활용 노쇠 수준 정의 절단값 등의 차이에 기인한 것으로 보인다. 현재까지 FI-LAB의 절단값은 일관되게 확립되어 있지 않으며, 연구 목적 및 환자군에 따라 다양하게 설정되고 있다[26-28]. 따라서, 향후 연구에서는 예후 예측력 기준 정립을 위한 절단값 확정 연구가 필요하며, 이를 바탕으로 객관적인 노쇠 평가 도구로 FI-LAB의 임상적 활용성을 높일 수 있다.

노쇠 수준은 연령 증가와 유의한 관련성을 보였으며, 이는 FI-LAB이 노쇠를 측정하는 타당한 도구임을 입증하였다[28]. 그러나 본 연구에서는 노쇠 수준이 높은 집단과 낮은 집단 간의 평균 연령에는 유의한 차이가 있었으나, 연령을 65세 이상과 미만으로 구분하였을 때는 집단 간 노쇠 수준 차이가 통계적으로 유의하지 않았다. 이는 Verschoor 등[29]의 연구에서 보고된 바와 같이 노쇠가 연령에 따라 선형적으로 증가할 뿐 아니라, 상대적으로 젊은 연령층에서도 다양한 건강 요인에 의해 나타날 수 있음을 뒷받침한다. 따라서 노쇠 평가는 연령만을 기준으로 하기보다는 생리적 예비능력, 만성질환, 영양 상태 등 다양한 요인을 종합적으로 고려하는 접근이 필요하다. 본 연구에서는 폐경 연령과 노쇠 수준 간에 유의한 차이가 나타나지 않아, 폐경 연령이 높을수록 노쇠 유병률이 유의하게 낮아지는 경향을 보고한 선행연구[30]와는 상이한 결과를 보였다. 본 연구는 특정 의료기관에서 부인암으로 수술을 받은 폐경 후 여성을 대상으로 한 후향적 단면 연구로, 진단 시기나 암의 진행 정도, 수술 전 전신 상태 등이 폐경 연령과 노쇠 수준 간의 관계에 영향을 미쳤을 가능성이 있다. 따라서 폐경 연령과 노쇠 간의 인과적 연관성을 보다 명확히 규명하기 위해서는 다양한 연령대와 건강 상태를 포함한 여성 집단을 대상으로 한 전향적 연구가 필요하다.

임상적 특성에서는 부인암 종류, 병기, ASA class, 마취 시간, 수술 시간에서 모두 노쇠 수준 간 유의한 차이가 나타났다. 특히 병기 진행에 따라 노쇠 수준이 높아지는 경향은 AlHilli 등[31]의 연구와 일치하며, 암의 진행에 따른 생리적 예비력 저하와 관련될 수 있다. 수술 전 ASA class 또한 FI-LAB과 유의한 관련을 보였으며, 이는 Pichatechaiyoot 등[32]의 연구에서 보고된 결과와 유사한 경향을 나타낸다. 따라서 수술 전 평가 시 ASA class와 함께 FI-LAB 평가를 병행하여 환자의 전신 상태를 보다 정밀하게 평가할 필요가 있다. 수술 시간 및 마취 시간의 증가 또한 노쇠 수준 증가와 관련되었으며, 이는 Chen 과 Qin [33]의 결과와 유사하다. 반면 본 연구에서 수술 방법은 노쇠 수준과 유의한 관련이 없었으나, 선행연구에서는 수술 방법과 노쇠 수준 간의 관련성을 보고한 바 있다. Wainger 등[34]의 연구에서는 개복수술을 받은 환자에서 노쇠군으로 분류된 경우가 복강경수술을 받은 환자보다 더 많아, 수술 방법에 따라 노쇠 수준에 차이가 있음을 확인하였다.

수술 후 건강 결과에서는 재원 기간과 수혈 유무에서 노쇠 수준에 따른 유의한 차이가 나타났다. 노쇠 수준이 높을수록 재원 기간이 길었으며, 이는 Xu 등[35]의 연구에서 보고된 결과와 유사하였다. 이와 같은 재원 기간의 증가는 노쇠 외에도 환자의 영양 상태, 수술 난이도, ASA class 등 복합적인 요인이 작용한 결과로 해석될 수 있다. 또한 수혈 유무에서도 노쇠 수준이 높은 집단에서 수혈을 받은 비율이 높았다. 이는 해당 집단이 빈혈이나 출혈에 더 취약했음을 시사하며, Cai 등[36]의 연구에서도 노쇠군에서 수혈 필요성이 더 높게 보고된 바 있다. 이러한 결과는 노쇠 환자가 수술 전 이미 빈혈 상태이거나 생리적 예비력이 저하되어 있어, 수술 중 출혈 위험과 회복 과정에서 외부적 보조가 더 많이 요구되기 때문으로 설명된다. 아울러 선행연구에서 수혈은 합병증 발생률 증가와 재원 기간 연장의 주요 요인으로 제시되어[37,38], 노쇠 수준이 수술 후 회복 경과에 중요한 영향을 미칠 수 있음을 보여준다.

한편, 수술 후 합병증 발생률은 노쇠 수준 간 유의한 차이를 보이지 않았으며, 이는 본 연구에서 발생 건수가 적고 후향적 자료 수집의 한계로 인해 일부 합병증이 누락되었을 가능성 등 다양한 제한점이 작용한 것으로 판단된다. 그러나 선행연구에서는 노쇠 환자의 합병증 발생 위험이 더 높다고 보고하고 있다. Nakhla 등[39]의 연구에서는 노쇠한 환자가 비노쇠 환자에 비해 호흡기(adjusted odds ratio [aOR] 2.6; *p* < .001), 신경학적(aOR 3.3; *p* < .001), 신장(aOR 2.0; *p* < .001), 감염성(aOR 3.2; *p* < .001) 합병증 발생 위험이 유의하게 증가하는 것으로 나타났다. 이러한 결과와 달리 본 연구에서 뚜렷한 차이가 나타나지 않은 것은 연구 설계의 후향성, 비교적 작은 표본 규모, 그리고 합병증 정의 및 기록 방식의 차이에서 기인할 수 있다. 따라서 노쇠 수준과 합병증 발생 간의 인과관계를 명확히 검증하기 위해서는 전향적이고 대규모 표본을 활용한 연구가 필요하다.

본 연구는 FI-LAB이 폐경 후 부인암 수술 환자의 수술 전 생리적 상태를 객관적으로 평가하고, 일부 수술 후 건강 결과를 예측하는 데 유용한 도구가 될 수 있음을 확인하였다. 첫째, 설문이나 관찰값이 아닌 일상적 진단검사 지표만으로 FI-LAB을 산출하여 노쇠를 정량화함으로써 주관적 편향을 최소화하고 임상현장에서의 활용 가능성을 높였다. 둘째, 연구 대상을 폐경 후 부인암 수술 환자로 한정함으로써 호르몬 상태와 동반질환 특성이 비교적 유사한 집단에서 노쇠와 수술 후 결과 간의 연관성을 평가하였다는 점에서, 다양한 암종이 혼합된 기존 연구와 차별성을 가진다. 그러나 본 연구에는 다음과 같은 제한점이 있다. 첫째, 단일 의료기관에서 후향적으로 수집된 의무기록을 기반으로 하였기 때문에 연구 결과를 일반화하는 데 제한이 있으며, 선택 편향의 가능성을 배제할 수 없다. 둘째, 노쇠에 영향을 미칠 수 있는 일상생활 수행 능력, 사회적 지지와 같은 사회심리적 요인과 수술 후 회복 과정에서의 간호중재 등은 포함되지 않아 결과 해석에 제한이 있다. 셋째, 본 연구에서 노쇠 측정 도구로 사용된 FI-LAB의 항목 중 Vitamin D는 최종 분석에 포함된 대상자에서 시행된 사례가 없어 분석에 반영되지 못하였으며, Vitamin B12 및 Folate (serum)의 경우에도 시행된 대상자 수가 적어 노쇠 수준을 충분히 반영하지 못하였다는 점 역시 제한점으로 고려된다. 넷째, FI-LAB을 활용하여 노쇠를 평가하였으나, 신체기능, 일상생활 수행능력, 임상적 노쇠 척도 등과 같은 임상적 평가가 병행되지 않아 대상자의 실제 노쇠 상태를 충분히 반영하는 데 한계가 있다. 또한 본 연구는 노쇠 수준과 일반적·임상적 특성에서 유의한 차이를 보인 변수들을 결과 분석에서 충분히 통제하지 못하였다는 제한점이 있으며, 이에 따라 노쇠 수준과 수술 후 건강 결과 간의 관련성에 대한 인과적 해석에는 주의가 필요하다. 그럼에도 불구하고, FI-LAB을 활용하여 폐경 후 부인암 환자의 수술 전 노쇠를 객관적으로 평가하고 노쇠 수준이 단기 회복 지표와 연관됨을 제시함으로써, 향후 연구 설계에서 고려해야 할 기초자료를 제공하였다는 점에서 의의가 있다. 이러한 제한점을 보완하고 FI-LAB의 임상 적용 가능성을 높이기 위해, 후속 연구를 제언한다. 첫째, 부인암 수술 환자의 수술 전 노쇠 수준을 간편하게 평가할 수 있도록 FI-LAB 항목을 간소화하고, 그 타당성을 검증하는 연구가 필요하다. 둘째, 다양한 혼란변수를 통제한 다기관 전향적 연구를 통해 FI-LAB의 예측력과 임상 유용성을 검증할 필요가 있다. 셋째, 임상 간호사가 수술 전 노쇠 상태를 정확히 파악하고 이에 따른 간호중재를 효과적으로 수행할 수 있도록, FI-LAB 활용 교육과 노쇠 관리 전략이 포함된 간호 교육 프로그램의 개발이 요구된다.

## 결론

본 연구는 폐경 후 부인암 수술 환자를 대상으로 FI-LAB을 활용하여 수술 전 노쇠 수준을 평가하고, 수술 후 건강 결과와의 차이를 분석함으로써 임상에서 노쇠 평가 도구의 활용 가능성을 고찰하고자 하였다. 연구 결과, 수술 전 노쇠 수준이 높을수록 수술 후 재원 기간이 길고 수혈이 필요한 경우가 많은 것으로 나타났으며, FI-LAB은 환자의 생리적 취약성을 반영하는 실용적인 평가 도구로 활용될 수 있음을 확인하였다. 특히 FI-LAB은 전자의무기록 기반의 자료를 활용하여 자동 산출이 가능하다는 점에서, 임상 현장에서 간호사가 활용하기에 효율적이라는 장점이 있다. 이러한 연구 결과는 수술 전 고위험군을 조기에 선별하고, 맞춤형 간호중재를 계획하는 데 실질적인 도움을 줄 수 있을 것으로 기대된다.

## Figures and Tables

**Table 1.** General and Clinical Characteristics of the Participants (N = 198)

Variables	M ± SD	n (%)
Age (years)	62.07 ± 8.49	
< 65		133 (67.2)
≥ 65		65 (32.8)
Age at menopause (n = 165)	51.38 ± 4.42	
Time since menopause (n = 165)	10.15 ± 8.25	
BMI (kg/m^2^)	24.33 ± 4.02	
Underweight (< 18.5)		9 (4.5)
Normal (18.5~22.9)		73 (36.9)
Overweight (23~24.9)		37 (18.7)
Obesity (≥ 25)		79 (39.9)
Educational level (n = 195)		
Elementary or less		55 (28.2)
Middle school		41 (21.0)
High school		72 (36.9)
Post-secondary (college or university)		27 (13.9)
Nutritional status		
Adequate		79 (39.9)
Low risk		115 (58.1)
Moderate risk		4 (2.0)
Type of gynecologic cancer^†^		
Endometrial cancer		85 (42.9)
Ovarian cancer		62 (31.3)
Cervical cancer		49 (24.8)
Vulvar cancer		2 (1.0)
Stage of cancer		
Ⅰ		139 (70.2)
Ⅱ		28 (14.2)
Ⅲ		24 (12.1)
Ⅳ		7 (3.5)
Neoadjuvant chemotherapy		
Yes		12 (6.1)
No		186 (93.9)
ASA classification		
Ⅰ		14 (7.1)
Ⅱ		148 (74.7)
Ⅲ		36 (18.2)
Duration of anesthesia (minutes)	204.06 ± 83.56	
Duration of surgery (minutes)	167.29 ± 80.38	
Operation method		
Laparoscopic surgery		86 (43.4)
Open surgery		75 (37.9)
Robot-assisted surgery		37 (18.7)

M = Mean; SD = Standard deviation; BMI = Body mass index; ASA classification = American Society of Anesthesiologists physical status classification. ^†^No patients with vaginal cancer were identified.

**Table 2.** Frailty Status According to the FI-LAB (N = 198)

Variable	M ± SD	n (%)
FI-LAB	0.25 ± 0.10	
Low frailty (< 0.25)		103 (52.0)
Moderate frailty (0.25~0.4)		79 (39.9)
High frailty (> 0.4)		16 (8.1)

FI-LAB = Frailty Index-Laboratory; M = Mean; SD = Standard deviation.

**Table 3.** Difference in Laboratory and Clinical Variables According to FI-LAB Categories (N = 198)

Categories	Low Frailty^a^ (n = 103)	Moderate Frailty^b^ (n = 79)	High Frailty^c^ (n = 16)	F or χ^2^ (*p*)	Post hoc test
General characteristics					
Age (years)	60.70 ± 6.52	63.09 ± 10.02	65.81 ± 10.09	3.56 (.030)	a < c^†^
< 65	74 (71.8)	50 (63.3)	9 (56.2)	2.43 (.298)	
≥ 65	29 (28.2)	29 (36.7)	7 (43.8)		
Age at menopause (n = 165)	51.46 ± 4.72	51.27 ± 4.18	51.33 ± 3.12	0.03 (.968)	
Time since menopause (n = 165)	9.02 ± 6.62	11.33 ± 9.94	12.78 ± 8.57	1.91 (.151)	
BMI (kg/m^2^)	24.20 ± 4.05	24.70 ± 4.06	23.30 ± 3.57	0.92 (.399)	
Underweight (< 18.5)	6 (5.8)	2 (2.5)	1 (6.2)		
Normal (18.5~22.9)	36 (34.9)	30 (38.0)	7 (43.8)	3.21 (.726)^†^	
Overweight (23~24.9)	22 (21.4)	12 (15.2)	3 (18.8)		
Obesity (≥ 25)	39 (37.9)	35 (44.3)	5 (31.2)		
Educational level (n = 195)					
Elementary or less	30 (29.4)	22 (28.6)	3 (18.8)	7.57 (.250)^†^	
Middle school graduate	17 (16.7)	19 (24.7)	5 (31.2)		
High school graduate	41 (40.2)	23 (29.8)	8 (50.0)		
Post-secondary (college or university)	14 (13.7)	13 (16.9)	0 (0.0)		
Nutritional status					
Adequate	46 (44.7)	30 (38.0)	3 (18.8)	5.59 (.159)^†^	
Low risk	56 (54.3)	47 (59.5)	12 (75.0)		
Moderate risk	1 (1.0)	2 (2.5)	1 (6.2)		
Clinical characteristics					
Type of gynecologic cancer					
Endometrial cancer	50 (48.6)	32 (40.5)	3 (18.8)	19.41 (< .001)^†^	
Ovarian cancer	20 (19.4)	35 (44.3)	7 (43.7)		
Cervical cancer	32 (31.0)	11 (13.9)	6 (37.5)		
Vulvar cancer	1 (1.0)	1 (1.3)	0 (0.0)		
Stage of cancer					
Ⅰ	79 (76.7)	54 (68.3)	6 (37.5)	18.59 (.001)^†^	
Ⅱ	14 (13.7)	9 (11.4)	5 (31.3)		
Ⅲ	5 (4.8)	15 (19.0)	4 (25.0)		
Ⅳ	5 (4.8)	1 (1.3)	1 (6.2)		
Neoadjuvant chemotherapy					
Yes	6 (5.8)	3 (3.8)	3 (18.8)	5.25 (.098)^†^	
No	97 (94.2)	76 (96.2)	13 (81.2)		
ASA classification					
Ⅰ	7 (6.8)	6 (7.6)	1 (6.2)	12.12 (.036)^†^	
Ⅱ	81 (78.6)	60 (75.9)	7 (43.8)		
Ⅲ	15 (14.6)	13 (16.5)	8 (50.0)		
Duration of anesthesia (minutes)	191.82 ± 74.16	210.80 ± 85.99	249.62 ± 111.18	3.85 (.023)	a < c^‡^
Duration of surgery (minutes)	155.36 ± 72.39	174.38 ± 82.15	209.06 ± 104.98	3.70 (.026)	a < c^‡^
Operation method					
Laparoscopic surgery	52 (50.5)	31 (39.2)	3 (18.8)	10.09 (.053)	
Open surgery	31 (30.1)	33 (41.8)	11 (68.7)		
Robot-assisted surgery	20 (19.4)	15 (19.0)	2 (12.5)		

Values are presented as the mean ± standard deviation or n (%). FI-LAB = Frailty Index–Laboratory; BMI = Body mass index; ASA classification = American Society of Anesthesiologists physical status classification. ^†^Fisher’s exact test; ^‡^Post hoc test: Tukey’s HSD.

**Table 4.** Difference in Postoperative Clinical Characteristics According to FI-LAB Level (N = 198)

Variables	Total (n = 198)	Low Frailty^a^ (n = 103)	Moderate Frailty^b^ (n = 79)	High Frailty^c^ (n = 16)	F or χ^2^ (*p*)	Post hoc test
Duration of PACU stay (minutes)	33.17 ± 8.44	33.38 ± 8.72	32.71 ± 8.65	33.44 ± 5.21	0.15 (.859)	
Postoperative LOS (days)	13.47 ± 8.96	12.48 ± 7.71	13.73 ± 7.52	19.25 ± 17.68	4.26 (.015)	a < c^‡^
Postoperative transfusion						
Yes	30 (15.2)	11 (10.7)	12 (15.2)	7 (43.8)	11.78 (.005)^†^	a < c^§^
No	168 (84.8)	92 (89.3)	67 (84.8)	9 (56.2)		
Postoperative complication						
Yes	28 (14.1)	16 (15.5)	9 (11.4)	3 (18.8)	0.94 (.538)^†^	
No	170 (85.9)	87 (84.5)	70 (88.6)	13 (81.2)		

Values are presented as the mean ± standard deviation or n (%). FI-LAB = Frailty Index–Laboratory; PACU = Post-anesthesia care unit; LOS = Length of stay. ^†^Fisher’s exact test; ^‡^Post hoc test: Tukey’s HSD; ^§^Post hoc test: Bonferroni.

## Data Availability

The data that support the findings of this study are available from the corresponding author upon reasonable request.

## Appendix A. Electronic Medical Record Items Included in the Study

**Table:**

Categories	Variable	EMR source	Observation period
General characteristics	Age	Nursing information survey	Admission date
	Age at menopause	Nursing information survey	Admission date
	Time since menopause	Nursing information survey	From the date of menopause of the day of surgery
	BMI	Nursing information survey	Admission date
	Education level	Nursing information survey	Admission date
	Nutritional status	Nutritional status record	Admission date
Clinical characteristics	Type of gynecologic cancer	Nursing information survey	Admission date
	Stage of cancer	Laboratory test results	Surgery date
	Neoadjuvant chemotherapy	Admission record	Admission date
	ASA classification	Anesthesia record	Surgery date
	Duration of anesthesia	Anesthesia record	Surgery date
	Duration of surgery	Operative record	Surgery date
	Operation method	Operative record	Surgery date
Frailty	FI-LAB	Laboratory test results	Pre-admission laboratory data
Postoperative health outcome	Duration of PACU stay	PACU nursing record	Surgery date
	Postoperative LOS	Discharge record	Surgery-to-discharge
	Transfusion	Transfusion record	Surgery-to-discharge
	Complication	Clinical observation record	Surgery-to-discharge

EMR = Electronic medical record; BMI = Body mass index; ASA classification = American Society of Anesthesiologists physical status classification; FI-LAB = Frailty Index–Laboratory; PACU = Post-anesthesia care unit; LOS = Length of stay.

## Appendix B. FI-LAB Variables and Corresponding Normal Ranges

**Table:**

Categories	Variable	Normal range	Unit	Type
Anemia	Hemoglobin	12~16	g/dL	whole blood
	Mean cell volume	80~96	fL	whole blood
	Red cell distribution width	11.6~14.6	%	whole blood
Inflammation	C-reactive protein	0~1	mg/dL	serum
	Segmented neutrophils percent	40~80	%	whole blood
Endocrine	Glucose	3.9~6.1	mmol/L	serum
	Glycohemoglobin levels	0~5.7	%	whole blood
Nutrients	Albumin	32~45	g/L	serum
	Folate, serum	3.1~17.5	ng/mL	serum
	Iron, refrigerated	10.7~26.9	μmol/L	serum
	Protein, total	60~78	g/L	serum
	Vitamin B12	118~701	pmol/L	serum
	Vitamin D	12~50	ng/mL	serum
Adiposity	Direct HDL-cholesterol	1.3+	mmol/L	serum
	Total cholesterol	3.88~6.47	mmol/L	serum
	Triglyceride	0.11~2.74	mmol/L	serum
Motor (indirectly)	Alkaline phosphatase	20~130	U/L	serum
	Phosphorus	0.74~1.52	mmol/L	serum
	Total calcium	2.3~2.74	mmol/L	serum
Others	Bicarbonate	21~28	mmol/L	serum
	Bilirubin, total	2~21	μmol/L	serum
	Blood urea nitrogen	2.9~8.2	mmol/L	serum
	Creatinine	45~90	μmol/L	serum
	Lactate dehydrogenase	100~190	U/L	serum
	Platelet count SI	150~450	1000cells/uL	whole blood
	Sodium	136~142	mmol/L	serum
	Uric acid	160~430	μmol/L	serum
	Blood pressure- diastolic	60~90	mmHg	vital sign
	Blood pressure- systolic	90~140	mmHg	vital sign
	Mean arterial pressure	70~105	mmHg	vital sign
	Pulse	60~99	Bpm	vital sign
	Pulse pressure	30~65	mmHg	vital sign

FI-LAB = Frailty Index–Laboratory; HDL = High-density lipoprotein; SI = Standard international.
